# Fungal community inside lichen: a curious case of sparse diversity and high modularity

**DOI:** 10.1186/s40793-023-00531-8

**Published:** 2023-10-03

**Authors:** Jiho Yang, Jung-Jae Woo, Seung-Yoon Oh, Wonyong Kim, Jae-Seoun Hur

**Affiliations:** 1 Korean Lichen Research Institute, Sunchon National University, 255 Jungang-ro, Suncheon, 57922 South Korea; 2 Department of Biology, Sunchon National University, 255 Jungang-ro, Suncheon, 57922 South Korea; 3 Department of Biology and Chemistry, Changwon National University, 20 Changwondaehak-ro, Changwon, 51140 South Korea

**Keywords:** Lichen, Endolichenic fungi, Community structure, Diversity, Network

## Abstract

**Background:**

Lichens represent not only the mutualism of fungal and photosynthetic partners but also are composed of microbial consortium harboring diverse fungi known as endolichenic fungi. While endolichenic fungi are known to exert a remarkable influence on lichen ecology through their crucial roles in nutrient cycling, bioprospecting and biodiversity, the enigmatic community structures of these fungal inhabitants remain shrouded in mystery, awaiting further exploration and discovery. To address knowledge gap, we conducted metabarcoding on two lichens using 18S gene amplification, *Dirinara applanta* and *Parmotrema tinctorum*, and compared their microbial communities to those found in the pine bark to which the lichens were attached. Our hypothesis was that the endolichenic communities would exhibit distinct diversity patterns, community structures, network structures, and specialist composition compared to the surrounding epiphytic community.

**Results:**

Our investigation has shed light on the clear demarcation between the endolichenic and epiphytic fungal communities, as they exhibit markedly different characteristics that set them apart from each other. This research demonstrated that the endolichenic communities are less diverse as compared to the epiphytic communities. Through community similarity analysis, we observed that two endolichenic communities are more similar to each other in terms of community composition than with the adjacent epiphytic communities. Moreover, we unveiled a striking contrast in the network structures between the endolichenic and epiphytic communities, as the former displayed a more modular and less nested features that is evocative of a potent host-filtration mechanism.

**Conclusions:**

Through our investigation, we have discovered that lichens harbor less intricate and interconnected fungal communities compared to the neighboring epiphytic environment. These observations provide valuable insights into the metagenomic architecture of lichens and offer a tantalizing glimpse into the unique mycobiome.

**Supplementary Information:**

The online version contains supplementary material available at 10.1186/s40793-023-00531-8.

## Introduction

Lichens are a symbiotic mutualism comprising lichen-forming fungi and their photosynthetic partners [1]. Mutualism in lichen symbiosis is considered to benefit both organisms, the carbon source of lichen-forming fungi is mainly supplied by photosynthetic partners [2] and lichen thalli physically protect photobionts from environmental stresses, such as UV irradiation and desiccation [3]. Recently, the lichen thallus has been identified a complex microbial consortium harboring diverse bacteria [4], yeasts [5], and filamentous fungi [6]. Endolichenic fungi (ELF) represent fungal group isolated from the lichen thallus [7]. ELF are considered similar to endophytic fungi because they do not exhibit disease symptoms [8] and produce diverse secondary metabolites [9]. Thus, ELF can be distinguished from lichenicolous fungi which are parasitic [10] and produce visible structures on lichen thalli [11]. Because various metabolites produced by ELF show bioactivity containing antibacterial and antifungal activity [9], these fungal residents are considered to enhance the host lichens’ immunity. Several microorganisms within the lichen thallus have been reported to affect the phenotypes [5, 12] and physiology [13, 14] of host lichens. Therefore, studying ELF’s community architecture is pivotal to understand lichen physiology.

Previous research about the community structure of ELF found that these fungi are a diverse group with a majority belonging to the Ascomycota phylum [15, 16]. The fact that the endophytic fungi and the ELF share similar taxonomic diversity suggests that they may have similar ecological roles and origins. Both abiotic (e.g., geography) and biotic (e.g., host lichen phylogeny) factors have been shown to influence the composition of ELF communities [15, 17]. To better understand how ELF communities differ from other fungal communities, it would be helpful to compare them with the fungi found in the surrounding environment of lichens. Chagnon et al. found that ELF communities tend to have a more nested structure compared to endophytic fungal communities [18]. In contrast to previous culture-based methods, recent studies utilizing advanced next-generation sequencing technology have revealed that the taxonomic diversity of ELF communities is markedly different. Metabarcoding analyses have identified Dothideomycetes and Eurotiomycetes as being particularly abundant in these communities. [16], whereas they were underrepresented in culture-dependent methods [19]. Distinguishing between ELF and lichenicolous fungi has become increasingly challenging due to the fact that a significant proportion of lichenicolous fungal sequences have been found in apparently healthy lichens [16]. As a result, there has been ongoing debate and disagreement over the classification and structure of ELF communities.

To gain a better understanding of the distinct structure of ELF communities, it would be valuable to conduct a comparative study between lichen communities and the surrounding environment using metabarcoding techniques. The goal of this study is to uncover the unique architecture of the ELF community in comparison to the neighboring epiphytic (EPF) community. We selected the EPF community as a suitable comparator to ELF because it is likely that the tree bark represents the original fungal pool from which the ELF community was established. This is supported by the fact that the tree bark is physically closest to the epiphytic lichen and existed prior to lichenization. To explore the differences between ELF and EPF communities, we analyzed the fungal communities within the epiphytic lichens *Dirinaria applanta* and *Parmotrema tinctorum*, as well as the EPF community of *Pinus thunbergii*, using metabarcoding techniques. Precisely, our aim was to investigate fundamental inquiries regarding the distinctions between ELF and EPF communities, focusing on aspects such as diversity patterns and network architecture. To accomplish this, we compared the fungal communities across six different aspects: alpha diversity, taxonomic composition, community similarity, network structure, core microbiome, and host specialists.

## Methods

### Study site and sampling process

**Fig. 1:**
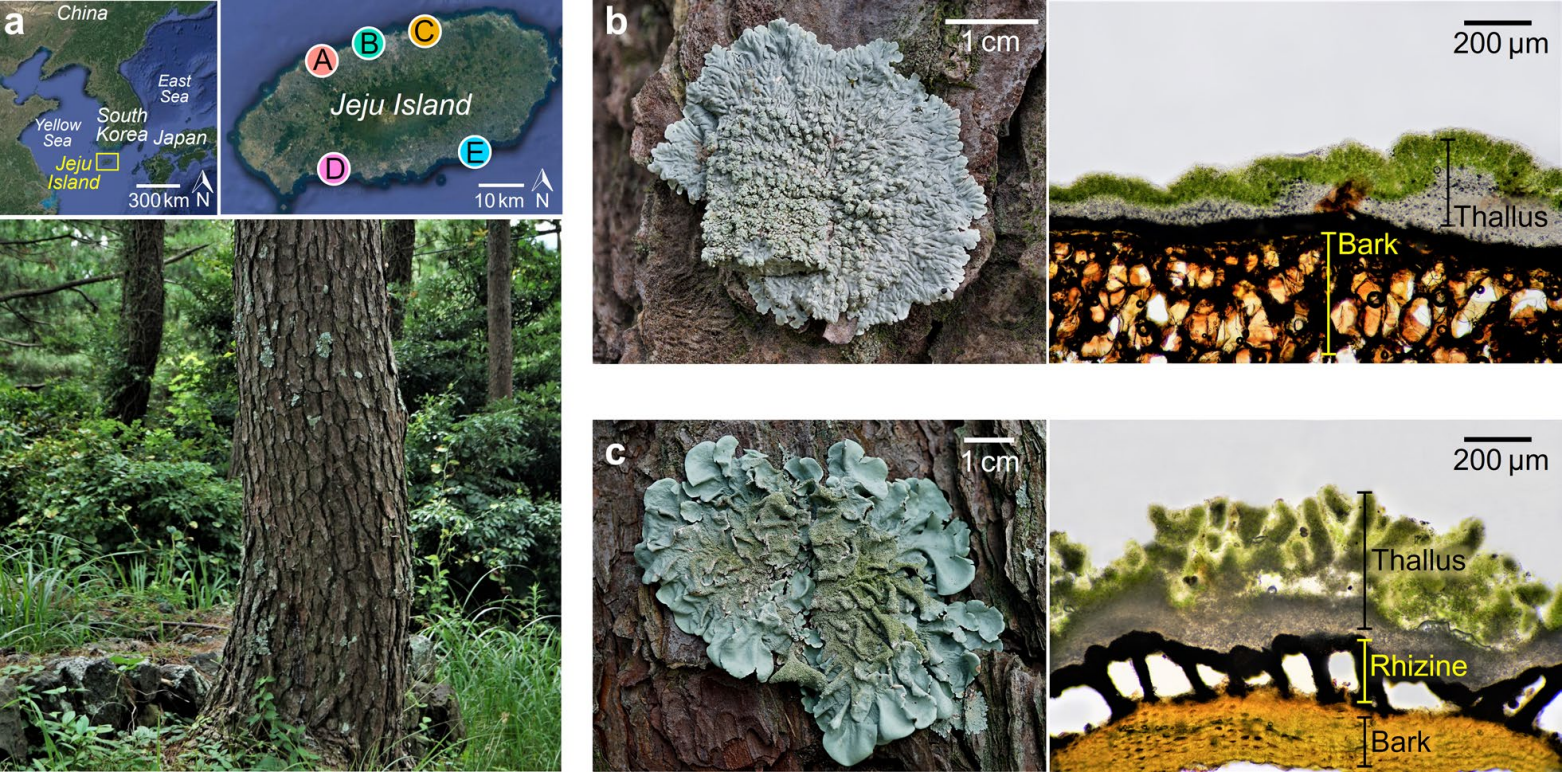
Collection information of this study. **a** Epiphytic lichens on *Pinus* bark located on Jeju Island, South Korea. Morphology of lichen thallus and microscopic view of **b** *Dirinaria applanata* and **c** *Parmotrema tinctorum*

We collected twenty five samples of *Pinus thunderbergii* bark, and the epiphytic lichen *Dirinaria applanta* and *Parmotrema tinctorum* from five sites (n = 75) on Jeju Island in the southern part of South Korea (Site A: 33° 30′ 27.5″ N, 126° 28′ 06.9″ E; Site B: 33° 28′ 43.2″ N, 126° 21′ 48.5″ E; Site C: 33° 31′ 05.0″ N, 126° 32′ 44.6″ E; Site D: 33° 14′ 16.1″ N, 126° 23′ 25.5″ E and Site E: 33° 16′ 23.8″ N, 126° 42′ 11.2″ E) (Fig. 1a). When we checked using the WorldClim database (https://www.worldclim.org/data/bioclim.html), the five collection sites did not show significant climatic differences (Additional file 1: Table S1). *Dirinaria* was strongly fixed to the *Pinus* bark (Fig. 1b), while the *Parmotrema* was relatively loosely attached to the substrate with its rhizine (Fig. 1c). The collected lichen thalli and the bark particles were cut into 1 cm^2^ pieces and their surface organic residues were removed using a syringe tip and running tap water. Subsequently, the surfaces of the lichen thalli were sterilized with 70% ethanol and 0.4% sodium hypochlorite (both for 90 s) as described by Yang et al. [20].

### Molecular and bioinformatics analyses

The bark and lichen segments were homogenized, and DNA was extracted using a PowerSoil DNA isolation kit (QIAGEN, CA, USA). We conducted a polymerase chain reaction (PCR) of the fungal ITS1 region [21] using the fungal specific primers ITS1F and ITS2 [22], ligated to Illumina sequencing adaptors. For each sample, PCR was performed three times using the AccuPower PCR PreMix kit (Bioneer, Daejeon, South Korea) under the following condition: 94 °C for 5 min, 25 cycles of 94 °C for 30 s, 55 °C for 30 s, and 72 °C for 40 s with a final extension at 72 °C for 10 min. The quality of the PCR products was evaluated in agarose gel and purified using the Expin PCR SV kit (GeneAll, Seoul, South Korea). A second PCR for barcoding was conducted to attach multiple index delimiters, as recommended by the Nextera XT index kit protocol (Illumina, CA, USA). The amplicon concentration was measured using NanoDrop2000 (Thermo Fisher Scientific, MA, USA) and pooled in equal molecular quantities.

Amplicon library sequencing was performed using the Illumina MiSeq platform (Macrogen, Seoul, South Korea). Raw sequences were processed using QIIME2 v.2021.4 [23] by demultiplexing and denoising reads following the DADA2 pipeline [24]. Taxonomic assignment was performed according to naïve Bayesian classifier guideline [25] using the UNITE fungi 99% operational taxonomic unit database [26]. Based on maximum likelihood method, a phylogenetic analysis was conducted using the q2-alignment plugin (https://docs.qiime2.org).

### Statistics and visualizations

The following analyses were performed using R v.3.5.3 [27]. For the statistical analysis, we conducted a Shapiro test [28] to check for data normality. Because the normality assumption was violated, we used the non-parametric Mann–Whitney U test [29] for pairwise comparisons and the Kruskal–Wallis test [30] followed by a Bonferroni correction [31] in multiple comparisons. All graphs were visualized using the “ggplot2” [32] and “ggpubr” [33] R packages. An amplicon sequence variant (ASV) [34] table was imported from QIIME2 into R using the “qiime2R package” (https://forum.qiime2.org) and rarefied to 3,000 sequences per sample using the “phyloseq” package [35]. The subsequent analyses, excluding the assessment of taxonomic composition were based on the rarefied ASV table.

The alpha diversity indices, Chao1 richness [36], Fisher’s alpha [37], Shannon’s diversity [38], and Shannon’s evenness [39] were calculated using the “vegan” package [40]. Community dissimilarity was calculated based on Bray–Curtis distance [41] using the “phyloseq” and “vegan” packages. Non-metric multidimensional scaling (NMDS) and principal co-ordinate analysis (PCoA) were performed using the “phyloseq” and “vegan” packages based on Bray–Curtis distance. The significance of NMDS was evaluated under stress value [42] and the standardized effect size of the PCoA was estimated using the *p* value of the permutation test [43] (n = 999). We statistically compared within-host (intra-host) similarity and between-host (inter-host) similarity using Bray–Curtis distance matrices by utilizing their average values. A hierarchical heatmap was constructed using the “pheatmap” package [44] with top the 100 most abundant ASV matrices. Ordination of the network structure and calculation of centrality indices were performed using the “qgraph” package [45] with a positive correlation cutoff of 0.2. We compared the fungal community network using the “RInSp” package [46]. We utilized seven ecological indices, namely *C*_*ws*_, Nestedness metric based on overlap and decreasing fill (NODF), Betweenness, Closeness, InDegree, OutDegree, and Expected influences. *C*_*ws*_ represents the level of modularity in the network, while NODF quantifies the extent of nestedness. Betweenness and Closeness indicate vectors that contain the betweenness centrality and closeness centrality of each node, respectively. InDegree and OutDegree indicate vectors that encompass the inward and outward degrees of each node. ExpectedInfluences indicates the sum of incoming or outgoing edge weights connected to a node. Identifying the core mycobiome [47] was conducted using the “microbiome” package [48] with a prevalence threshold of 0.2. Linear discriminant analysis (LDA) [49] was performed using the “microbial” [50] and “microbiome” packages, with cutoffs of LDA scores > 2 and *p* < 0.05. Chi-squared tests [51] and volcano plot visualizations were constructed to identify differently abundant taxa by host type using the “DESeq2” [52] and “EnhancedVolcano” [53] packages, and host specialist ASVs were selected by satisfying the thresholds of log_2_ fold change > 2 and *p* < 0.05.

## Results

### Summary of generated fungal sequencing data

**Fig. 2:**
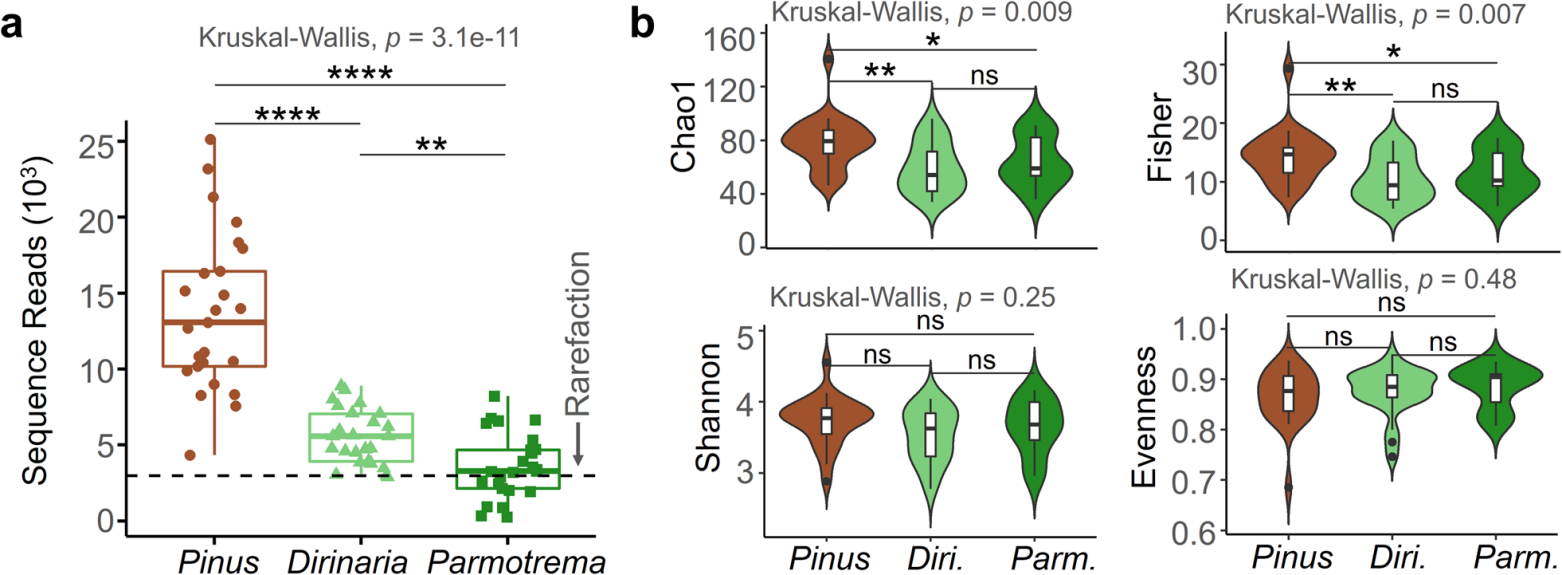
Alpha diversity and taxonomic composition of the fungal communities. **a** Sequence reads of the fungal communities (mean ± SEM, ^****^p < 0.001, ^**^p < 0.01). The dashed line indicates the depth of sequence rarefaction. **b** Alpha diversity of the fungal communities were calculated using four indices: Chao1 richness, Fisher’s alpha, Shannon’s diversity, and Shannon’s evenness (mean ± SEM, ^**^p < 0.01, ^*^p < 0.05, ns not significant)

After denoising and filtering out the host lichen fungal sequences, the ITS amplicon data yielded 571,273 total fungal sequences in the *Pinus* bark and lichen samples. The number of sequence reads were significantly different by fungal host type; the number of EPF sequence reads of *Pinus* were 13,700 ± 5,169, which is higher than those of the ELF sequences of *Dirinaria* (5,520 ± 1,811) and *Parmotrema* (3,630 ± 2,148). For a comparative analysis of the fungal communities, a rarefaction process [54] was implemented, remaining at 3,000 sequences per community (Fig. 2a). In this process, two ELF communities of *Dirinaria* and ten *Parmotrema* communities were excluded from the further analyses because they were below this cutoff values.

### Contrasting fungal diversity in the pine barks and lichens

The alpha diversity of the ELF and EPF communities was assessed using four diversity indices (Fig. 2b). Results showed that Chao1 richness and Fisher’s alpha were higher in the EPF communities of pine bark than in the ELF communities of *Dirinaria* and *Parmotrema*, but not significantly different between the two ELF communities. Shannon’s diversity and evenness did not vary significantly between host types. Thus, the data suggested that fungal diversity was lower in lichens than in pine bark.

**Fig. 3:**
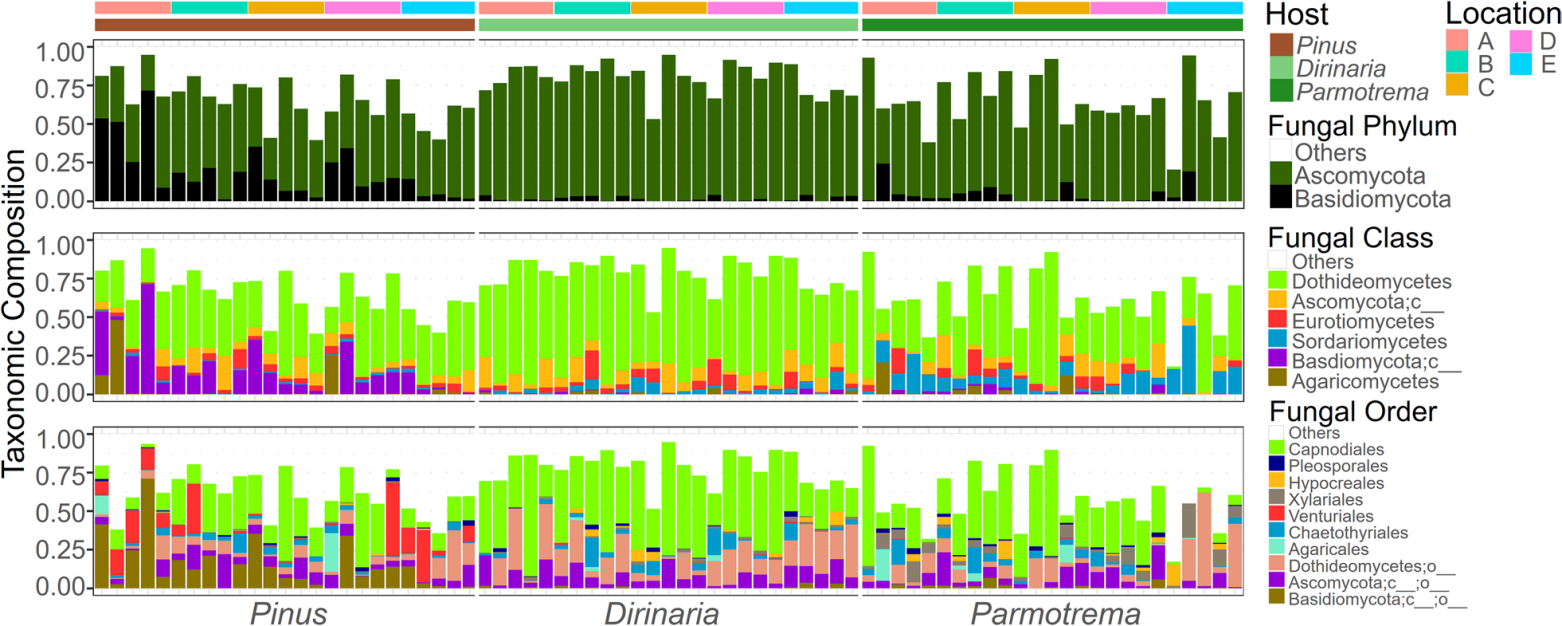
Taxonomic composition of the fungal communities. The taxonomic composition of the fungal community was presented at three hierarchical levels: phylum, class, and order. The topmost row represents location, while the second row represents host type

Taxonomic compositions of the ELF and EPF communities were distinguishable, with differences in Basidiomycota frequency being particularly pronounced (as shown in Fig. 3). At the phylum level, Ascomycota was dominant in both ELF communities, comprising 46.8% of the EPF communities and showing strong dominance in *Dirinaria* and *Parmotrema*. In contrast, Basidiomycota accounted for 27.9% of the EPF communities and were less abundant in the ELF communities. At the class level, the Ascomycota-dominant pattern diverged in the two ELF communities, with Dothideomycetes being the dominant fungal class in both communities. Sordariomycetes were more abundant in *Parmotrema* than in *Dirinaria*. The most abundant order in all fungal communities was Capnodiales.

### Greater resemblance between the ELF communities than to the EPF community

**Fig. 4:**
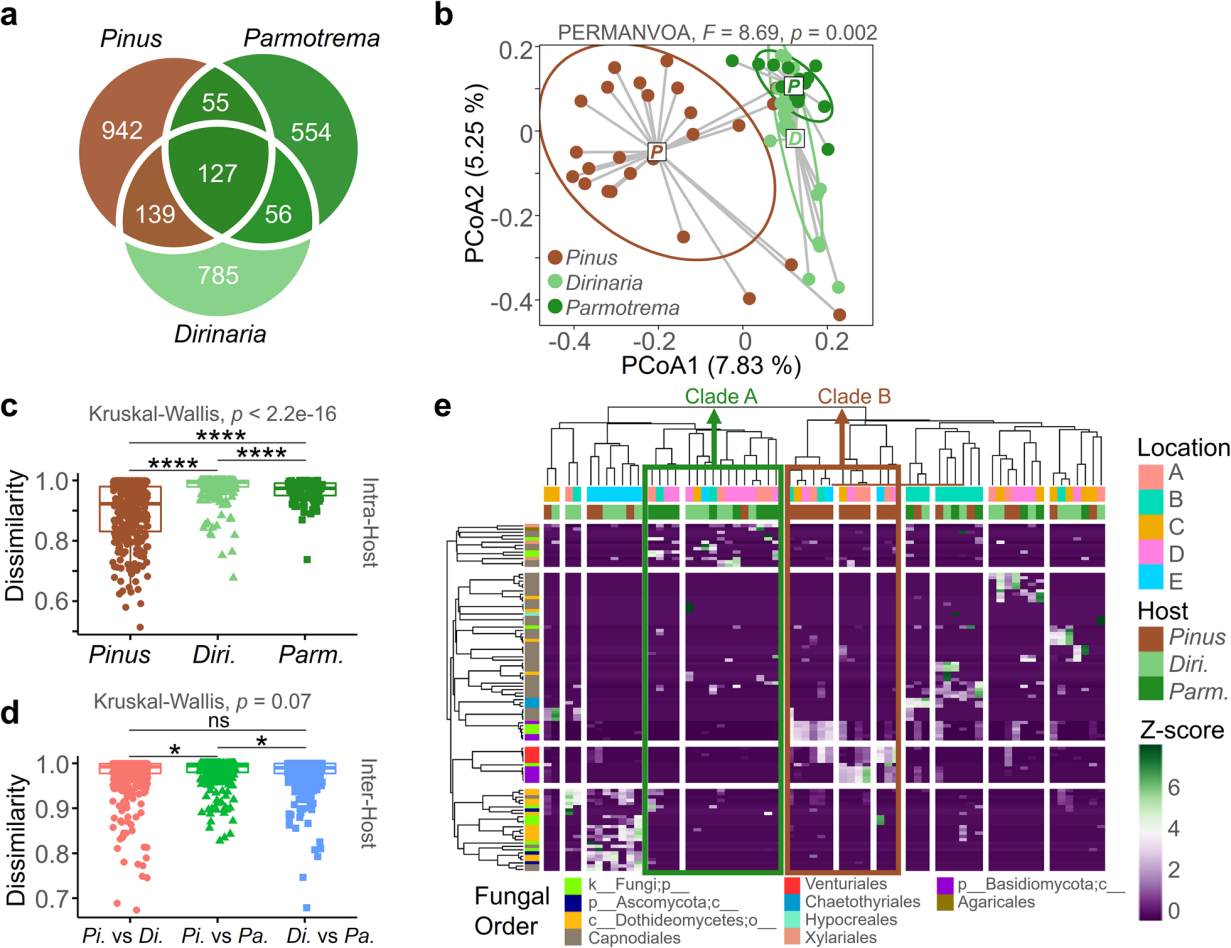
Beta diversity of the fungal communities. **a** Venn-diagram showing the shared ASV numbers of the fungal communities. **b** Principal component analysis (PCoA) based on Bray–Curtis distance including ellipse of 95% confidence interval. Distance to centroid of PCoA representing intra-host variance of the fungal communities (mean ± SEM, ^**^p < 0.01, ^*^p < 0.05, ns not significant). **c** Intra- and inter-host dissimilarity based on Bray–Curtis distance (mean ± SEM, ^****^p < 0.001, ^*^p < 0.05, ns not significant). **d** Hierarchical heatmap based on the Bray–Curtis distance of the fungal communities’ 100 most abundant ASVs

The three fungal communities shared a total of 127 ASVs in common, and a significant proportion of the EPF ASVs were also found in the ELF communities, with 24% in *Dirinaria* and 23% in *Parmotrema* mycobiome (Fig. 4a). The fungal communities were clearly separated by host type, as shown by PCoA (Fig. 4b), and not by geographical distance (Fig. S1). The two ELF communities were more similar to each other than to the EPF communities. The EPF communities displayed significantly lower intra-host dissimilarity compared to the ELF communities of *Dirinaria* and *Parmotrema* (Fig. 4c).

It is worth mentioning that the two ELF communities exhibited a higher degree of similarity to each other than to the EPF community, with the EPF community displaying a higher resemblance to the ELF community of *Dirinaria* (*Pi*. vs. *Di*.: 0.978 ± 0.041) than to that of *Parmotrema* (*Pi*. vs. *Pa*.: 0.983 ± 0.030) (Fig. 4c). The hierarchical heatmap analysis indicated that the fungal communities were primarily clustered according to their host type rather than geographical location (Fig. 4d). Specifically, the two ELF communities were grouped together in Clade A, characterized by a high abundance of Capnodiales. In contrast, the EPF communities were grouped in Clade B, marked by abundant unclassified Basidiomycota and Venturiales. Taken together, our findings suggest that the two ELF communities had comparable structures, and that the EPF community was more akin to the ELF community of *Dirianria* than to that of *Parmotrema*.

### Less nestedness and enhanced modulararity in the ELF community network

**Fig. 5:**
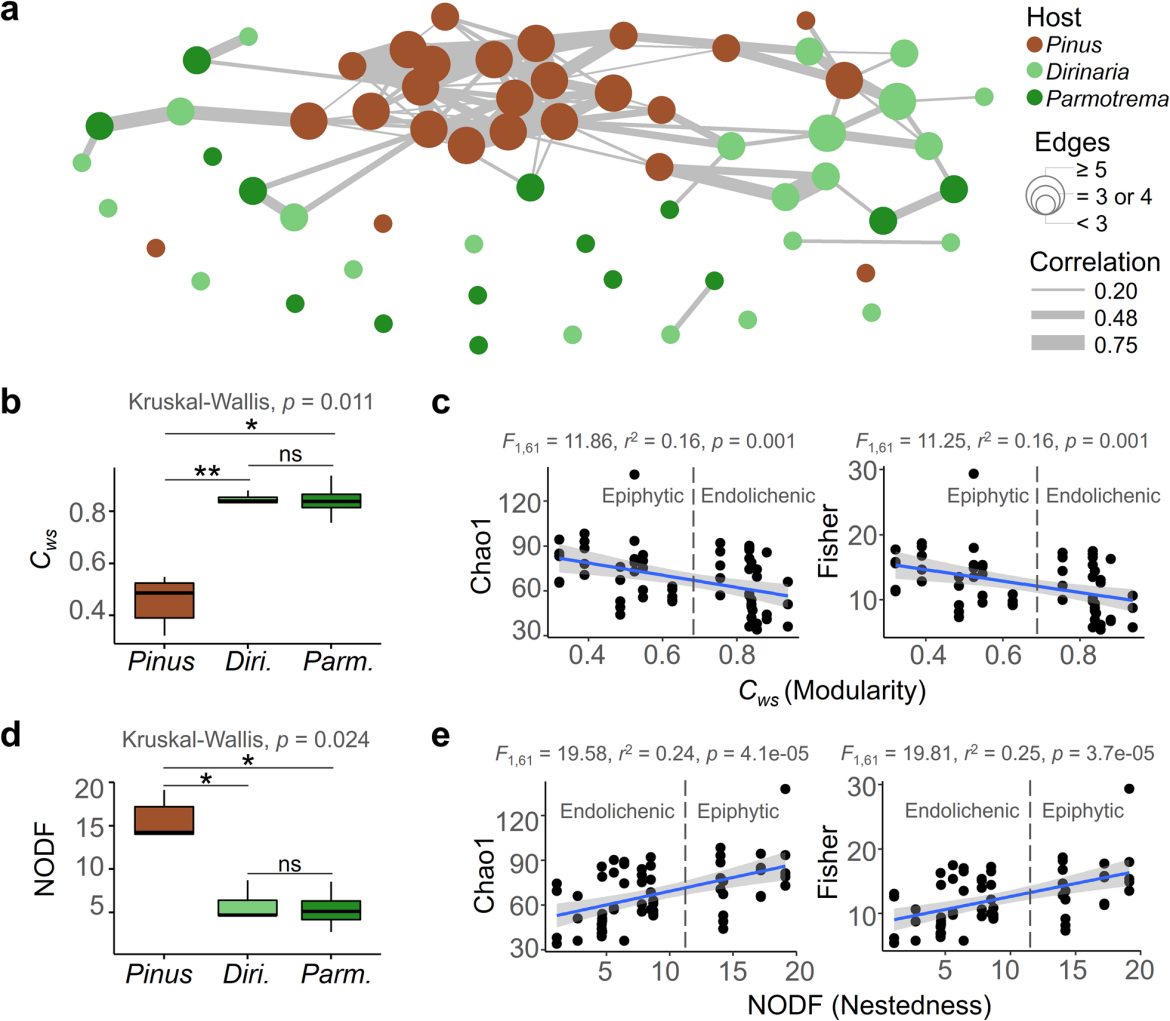
Network structure of the fungal communities. **a** Correlation web of the fungal communities. Edge size represents the number of nodes and node thickness indicates the degree of Pearson correlation. **b** Modularity (*C*_*ws*_) of the fungal communities (mean ± SEM, ^**^p < 0.01, ^*^p < 0.05, ns not significant). **c** Linear regression of relationship between fungal richness (Chao1), diversity (Fisher) and modularity of the fungal communities. **d** Nestedness (nestedness metric based on overlap and decreasing fill, NODF) of the fungal communities (mean ± SEM, *p < 0.05, ns not significant). **e** Linear regression of the relationship between fungal richness (Chao1), diversity (Fisher) and NODF

The co-occurrence network plot demonstrated that the ELF communities exhibited less nestedness compared to the EPF communities (Fig. 5a). The number of nodes in the EPF communities was higher compared to the ELF communities. The vectors containing betweenness and closeness of each node were significantly higher in the EPF communities than in the ELF communities (Additional file 1: Fig. S2a). The inward and outward degree vectors of each node were also higher in the EPF communities than in the ELF communities (Additional file 1: Fig. S2b). Moreover, the expected influences which indicate the sum of incoming or outgoing edge weights connected to a node were also significantly higher in the EPF communities than in the ELF communities (Additional file 1: Fig. S2c).

The network structure of the ELF and the EPF communities were highly distinguished. The ELF communities exhibited a higher weighted clustering coefficient *C*_*ws*_, which represents the degree of modularity [55] than the EPF communities (Fig. 5b). The *C*_*ws*_ showed a negative relationship with species richness (Chao1) and diversity (Shannon) (Fig. 5c). The nestedness of the community structure, as measured by the nestedness metric based on overlap and decreasing fill (NODF), [55] was higher in the EPF communities than in the ELF communities (Fig. 5d). Notably, the *C*_*ws*_ and the NODF of two ELF communities had non-significant differences (Figs. 3b and d). In contrast to the *C*_*ws*_, the NODF had a positive correlation with species richness and diversity (Fig. 5e). Taken together, the ELF communities were clearly distinguished to the EPF community in terms of their more modular and less nested features.

### Core mycobiomes of the fungal communities

**Fig. 6:**
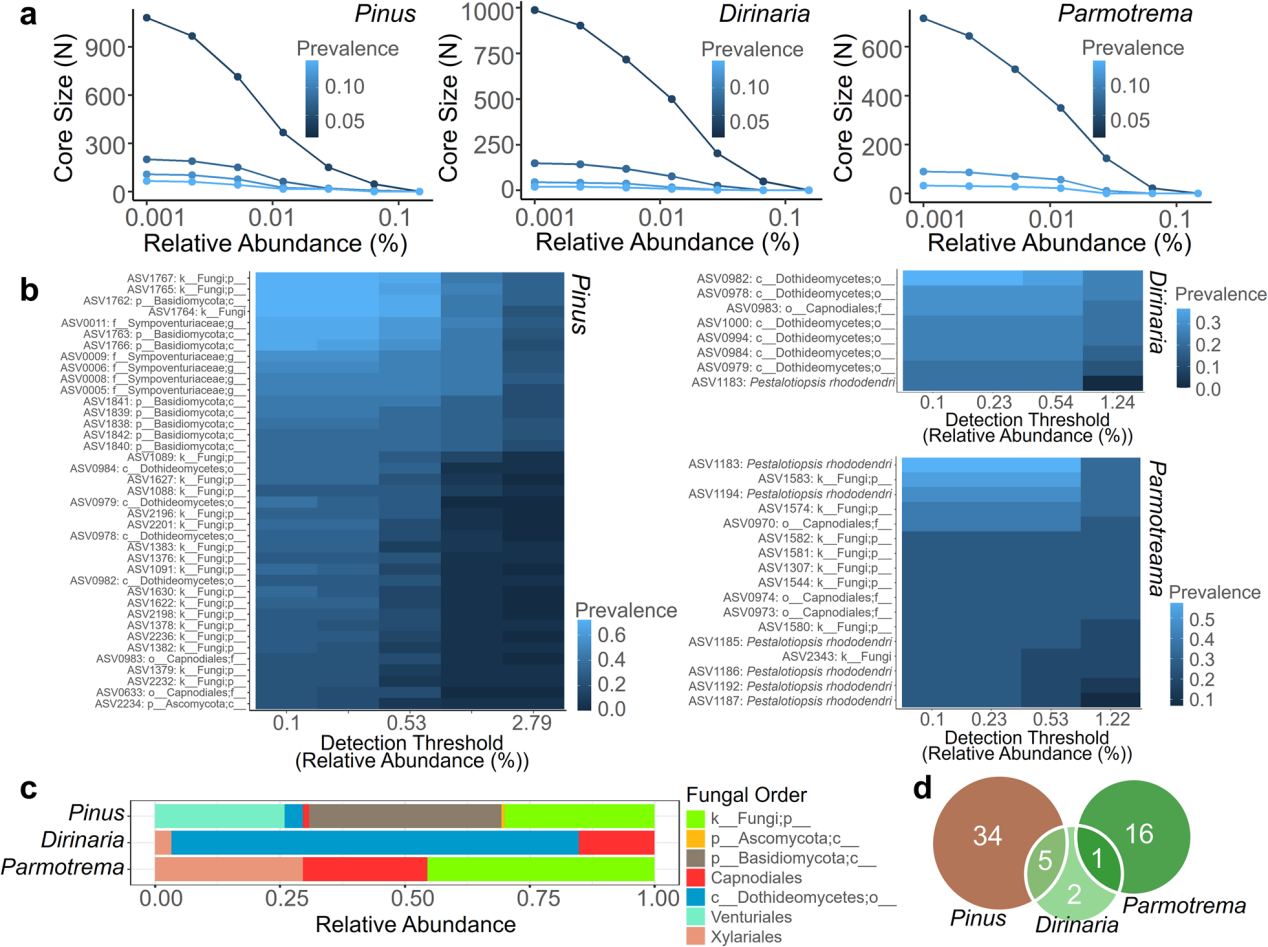
Core mycobiome of the fungal communities. **a** Line plot of the relationship between size of the fungal communities’ core mycobiome, prevalence and relative abundance. **b** Profile of the core mycobiomes with their detection threshold and prevalence. **c** Taxonomic composition of the core mycobiomes of the fungal communities. **d** Venn diagram showing shared number of core mycobiome of the fungal communities

To assess the variation in core structures between the EPF and ELF fungal communities, we determined and contrasted the core mycobiomes. Increasing the threshold for relative abundance and prevalence resulted in a reduction in the size of the core mycobiome of the fungal communities. (Fig. 6a). At a threshold of 0.2 prevalence, the EPF communities had a larger core mycobiome consisting of 39 ASVs, whereas the core mycobiomes of the ELF communities of *Dirinaria* and *Parmotrema* were smaller, with 8 and 17 ASVs, respectively. (Fig. 6b). The taxonomic compositions of the core mycobiome in the fungal communities showed significant differences between the host types (Fig. 6c). The dominant taxonomic groups in the core mycobiomes of the EPF and ELF communities varied depending on the host type. Unclassified Basidiomycota, unclassified Fungi, and Venturiales were the most abundant in the EPF communities, while unclassified Dothideomycetes, Capnodiales, and Xylariales were abundant in the ELF communities of *Dirinaria*. In the core mycobiome of *Parmotrema*, unclassified Fungi, Xylariales, and Capnodiales were dominant. The genus *Pestalotiopsis* was identified as a significant member of the ELF community in the core mycobiome analysis. Importantly, *Pestalotiopsis* holds core membership status within both *Dirinaria* and *Parmotrema* lichens, which stands in contrast to its absence in EPF. The Venn diagram revealed that *Pinus* bark and *Dirinaria* shared five core fungal ASVs, indicating a partial overlap (Fig. 6d). An intriguing finding was that there were no shared core fungal members between the EPF community and the ELF community of *Parmotrema*, supporting the conclusion from the community similarity analysis that the mycobiome of *Dirinaria* was more similar to that of the EPF community than that of *Parmotrema*.

### Identification of ELF and EPF specialists

**Fig. 7:**
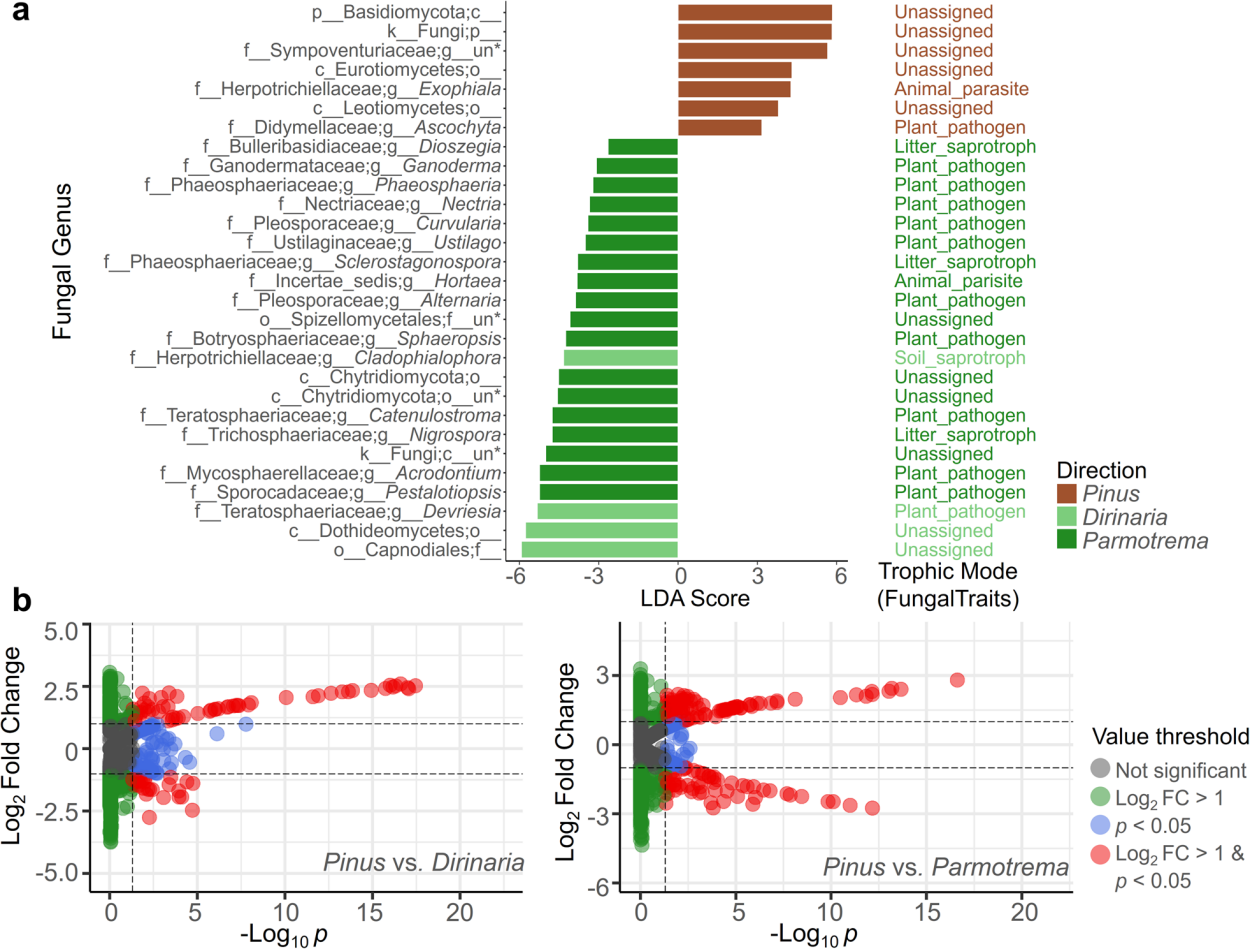
Profile of fungal host specialists. **a** Fungal specialist identification based on linear discriminant analysis. Trophic modes based on FungalTraits database of fungal host specialists are listed. **b** Volcano plot based on a chi-squared test of pairwise comparison between the endolichenic and epiphytic fungal communities

We employed linear discriminant analysis (LDA) to identify host specialists. We observed host-specific ELF and EPF at the genus level (shown in Fig. 7a). Several genera including unclassified Basiomycota were found to be EPF specialists, while others showed high LDA scores in the ELF communities of *Dirinaria* and *Parmotrema*. The trophic mode analysis based on FungalTraits [56], showed that fungal group annotated to plant pathogen were more abundant in the ELF communities than in the EPF communities.

To further analyze the differences between the fungal communities, pairwise comparisons were conducted using a chi-squared test (Fig. 7b). The results showed that in the comparison between the EPF and *Dirinaria* communities, there were 52 EPF and 20 ELF specialists identified (Additional file 1: Tables S2 and S3). In the comparison between EPF and ELF communities of *Parmotrema*, 64 EPF and 42 ELF specialists were observed. These findings were consistent with the LDA analysis and showed that the abundance of plant-pathogen fungi was higher in the ELF communities than in the EPF communities.

## Discussion

While the diversity and structure of fungal communities within lichen thalli play a critical role in their ecological functioning [11, 17], they have been relatively understudied. Furthermore, the comparative analysis of these communities with neighboring EPF communities has not been thoroughly explored. This study addresses this gap by conducting a comparative analysis of the mycobiome structure within lichen and nearby EPF communities, focusing on several aspects of diversity and network analysis.

The fungal communities of EPF were found to exhibit higher richness and diversity compared to ELF communities, suggesting that epiphytic fungi might serve as a potential source of fungal diversity in lichen thalli attached to the bark. Moreover, the considerable overlap of ASVs between the EPF and ELF communities further strengthens this proposition. The structure of ELF communities appeared to be influenced by the filtering effect of lichen hosts, as their taxonomic compositions were more strongly associated with host identity than with geographical location. The occurrence of Basidiomycetes in the EPF communities was a frequent finding, corroborating previous reports on the occurrence of wood-decay fungi on *Pinus* bark [57]. The low abundance of Basidiomycota in the ELF communities could be due to the presence of antifungal metabolites such as atranorin in *D. applanta* [58] and chloroatranorin in *P. tinctorum* [59], which inhibit the growth of saprotrophs within lichen thallus. Interestingly, the most abundant taxa in the ELF communities, Capnodiales are known for their ability to resist biodeterioration through highly-melanized hyphae, as reported in a previous study [60]. Capnodiales fungi may therefore be able to successfully complete their life cycle within lichen thallus despite the presence of antifungal metabolites.

The hypothesis of host-filtration was supported by community similarity analyses, which showed that the ELF communities of two different host lichens were more similar to each other than to the EPF communities. Notably, the ELF of *Dirinaria* were found to be more similar to the EPF communities than those of *Parmotrema*, possibly due to the physical distance between the lichens and the bark. This is consistent with a previous study by Noh et al. [61] which showed that even micro-scale distance could lead to dissimilarity in fungal communities; *P. tinctorum* is relatively loosely attached to *Pinus* bark with its rhizine. Furthermore, this might be attributed to the similarity in physical properties of the hosts. The resemblance between *Dirinaria* ELF and EPF could potentially be elucidated through subsequent experimental approaches.

The network analysis revealed that the ELF communities exhibited a more modular and less nested architecture compared to the EPF communities. A higher modularity suggests that the ELF communities are more robustly sorted out by the host lichen compared to the pine bark, as discussed by Chagnon et al. [18]. This speculation is further supported by the observed inverse correlation between fungal diversity and modularity. These findings differ from those of previous research that examined the differences between fungal communities in epiphytic and endophytic environments [62]. It can be inferred that the filtration of fungal communities by the host lichens is more prominent compared to that of phyllosphere.

The ELF communities tend to have a lower number of core mycobiome compared to the EPF communities. According to a previous study, the core mycobiome of ELF communities was found to be largely dominated by fungi from the Capnodiales order [16]. In line with the community similarity analysis, the ELF communities of *Dirinaria* were found to have a higher level of core mycobiome overlap with the EPF communities compared to the ELF communities of *Parmotrema*. The core member of ELF, *Pestalotiopsis*, a fungus known for its role as both a plant endophyte and a pathogen was observed in the *Parmotrema* ELF communities. This observation was consistent with the finding that the *Parmotrema* ELF communities had the most diverse plant pathogen fungi, followed by the *Dirinaria* ELF communities and the EPF communities. These results suggest that ELF communities are similar to phyllosphere fungal communities, as previously proposed [11].

## Conclusion

Our analysis compared the structure of the ELF communities with that of their neighboring environments, the EPF communities. We found that the ELF community had a less diverse, less nested, and more modular structure than the EPF community. These features suggest that the distinctive architecture of the mycobiome in lichens may be shaped by strong host-filtration processes. Additionally, we found that the ELF community exhibited similarities to phyllosphere fungal communities, with a high abundance of plant pathogen fungi. While we have provided insights into the structure of ELF communities, many questions remain unanswered, such as the origin of ELF, its ecological role, and the interactions between ELF and host lichens. Future studies are needed to explore the ecological niche of ELF.

### Supplementary Information

**Additional file 1**. **Fig. S1** Community similarity ordination based on geographical distance. The ordinations are visualized in two approaches: **a** non-metric multidimensional scaling and **b** principal co-ordinates analysis based on Brat-Curtis distance. Ellipses indicate the 95% confidence interval of group variance. **c** PCoA plots showing ELF community variability between the two host lichens from same site.** Fig. S2** Centrality indices of the fungal communities. **a** Betweenness and Closeness indicate vectors containing the betweenness and closeness of each node. **b** InDegree and OutDegree indicate vectors containing the inward and outward degree of each node. **c** Expected influences indicates the sums of incoming or outgoing edge weights connected to a node. Mean ± SEM, ^****^p < 0.001, ^**^p < 0.01, ns not significant.

## Data Availability

All data are accessible on Sequence Read Archive (PRJNA932935), National Center for Biotechnology Information.

## Abbreviations

ELF: Endolichenic fungi
EPF: Epiphytic fungi
PCR: Polymerase chain reaction
ASV: Amplicon sequence variant
NMDS: Non-metric multidimensional scaling
PCoA: Principal co-ordinates analysis
LDA: Linear discriminant analysis
