# Supplementary material for: Fungal community inside lichen: a curious case of sparse diversity and high modularity
Source: Environ Microbiome. 2023 Oct 3;18:73. doi: 10.1186/s40793-023-00531-8 (PMC10548754; doi:10.1186/s40793-023-00531-8)
Supplement: Supplementary file 1 — Additional file 1. Fig. S1 Community similarity ordination based on geographical distance. The ordinations are visualized in two approaches: a non-metric multidimensional scaling and b principal co-ordinates analysis based on Brat-Curtis distance. Ellipses indicate the 95% confidence interval of group variance. c PCoA plots showing ELF community variability between the two host lichens from same site. Fig. S2 Centrality indices of the fungal communities. a Betweenness and Closeness indicate vectors containing the betweenness and closeness of each node. b InDegree and OutDegree indicate vectors containing the inward and outward degree of each node. c Expected influences indicates the sums of incoming or outgoing edge weights connected to a node. Mean ± SEM, ****p < 0.001, **p < 0.01, ns not significant. [file 40793_2023_531_MOESM1_ESM.zip › Table S1.docx]

**Table S1** Climatic data of the collection sites

| **WorldClim Variables** | **LOC A** | **LOC B** | **LOC C** | **LOC D** | **LOC E** |
| --- | --- | --- | --- | --- | --- |
| Annual Mean Temperature | 131 | 153 | 151 | 141 | 152 |
| Mean Diurnal Range (Mean of monthly (max temp - min temp)) | 66 | 63 | 65 | 67 | 71 |
| Isothermality (BIO2/BIO7) (×100) | 23 | 23 | 24 | 24 | 26 |
| Temperature Seasonality (standard deviation ×100) | 7320 | 7258 | 7258 | 7205 | 7149 |
| Max Temperature of Warmest Month | 273 | 295 | 292 | 282 | 293 |
| Min Temperature of Coldest Month | -3 | 26 | 23 | 7 | 20 |
| Temperature Annual Range (BIO5-BIO6) | 276 | 269 | 269 | 275 | 273 |
| Mean Temperature of Wettest Quarter | 221 | 249 | 241 | 229 | 240 |
| Mean Temperature of Driest Quarter | 61 | 63 | 82 | 115 | 85 |
| Mean Temperature of Warmest Quarter | 227 | 249 | 247 | 236 | 247 |
| Mean Temperature of Coldest Quarter | 38 | 63 | 60 | 50 | 62 |
| Annual Precipitation | 1534 | 1413 | 1532 | 1514 | 1760 |
| Precipitation of Wettest Month | 229 | 231 | 253 | 218 | 273 |
| Precipitation of Driest Month | 45 | 44 | 45 | 45 | 49 |
| Precipitation Seasonality (Coefficient of Variation) | 53 | 55 | 56 | 51 | 53 |
| Precipitation of Wettest Quarter | 667 | 637 | 696 | 640 | 784 |
| Precipitation of Driest Quarter | 175 | 169 | 177 | 174 | 193 |
| Precipitation of Warmest Quarter | 645 | 637 | 687 | 610 | 723 |
| Precipitation of Coldest Quarter | 175 | 169 | 177 | 176 | 198 |
