# Supplementary material for: Fungal community inside lichen: a curious case of sparse diversity and high modularity
Source: Environ Microbiome. 2023 Oct 3;18:73. doi: 10.1186/s40793-023-00531-8 (PMC10548754; doi:10.1186/s40793-023-00531-8)
Supplement: Supplementary file 1 — Additional file 1. Fig. S1 Community similarity ordination based on geographical distance. The ordinations are visualized in two approaches: a non-metric multidimensional scaling and b principal co-ordinates analysis based on Brat-Curtis distance. Ellipses indicate the 95% confidence interval of group variance. c PCoA plots showing ELF community variability between the two host lichens from same site. Fig. S2 Centrality indices of the fungal communities. a Betweenness and Closeness indicate vectors containing the betweenness and closeness of each node. b InDegree and OutDegree indicate vectors containing the inward and outward degree of each node. c Expected influences indicates the sums of incoming or outgoing edge weights connected to a node. Mean ± SEM, ****p < 0.001, **p < 0.01, ns not significant. [file 40793_2023_531_MOESM1_ESM.zip › Table S2.docx]

**Table S2** Result of a chi-squared test of the pairwise comparison between the endolichenic fungal communities of *Dirinaria* and epiphytic fungal communities

| **Abundant in** | **ASV no.** | **Taxa** | **Trophic Mode** | **Log_2_ FC_­_^*^** | **LSE^*^** | ***p* value** |  |
| --- | --- | --- | --- | --- | --- | --- | --- |
| *Pinus* Bark | ASV1763 | unclassified Basidiomycota | unassigned | 2.603 | 0.319 | 2.49E-17 | |
| *Pinus* Bark | ASV1766 | unclassified Basidiomycota | unassigned | 2.553 | 0.32 | 9.88E-17 | |
| *Pinus* Bark | ASV1841 | unclassified Basidiomycota | unassigned | 2.529 | 0.325 | 3.54E-18 | |
| *Pinus* Bark | ASV0005 | unidentified Sympoventuriaceae | unassigned | 2.522 | 0.324 | 4.84E-17 | |
| *Pinus* Bark | ASV1839 | unclassified Basidiomycota | unassigned | 2.477 | 0.325 | 6.86E-17 | |
| *Pinus* Bark | ASV1838 | unclassified Basidiomycota | unassigned | 2.459 | 0.326 | 1.01E-17 | |
| *Pinus* Bark | ASV0008 | unidentified Sympoventuriaceae | unassigned | 2.421 | 0.325 | 1.82E-16 | |
| *Pinus* Bark | ASV1842 | unclassified Basidiomycota | unassigned | 2.412 | 0.326 | 8.71E-18 | |
| *Pinus* Bark | ASV1840 | unclassified Basidiomycota | unassigned | 2.346 | 0.326 | 1.19E-15 | |
| *Pinus* Bark | ASV1762 | unclassified Basidiomycota | unassigned | 2.339 | 0.322 | 1.41E-14 | |
| *Pinus* Bark | ASV0011 | unidentified Sympoventuriaceae | unassigned | 2.289 | 0.324 | 2.40E-14 | |
| *Pinus* Bark | ASV1767 | unclassified Fungi | unassigned | 2.284 | 0.322 | 5.43E-14 | |
| *Pinus* Bark | ASV2435 | unclassified Fungi | unassigned | 2.245 | 0.318 | 0.0004 | |
| *Pinus* Bark | ASV2585 | unclassified Fungi | unassigned | 2.237 | 0.301 | 0.0137 | |
| *Pinus* Bark | ASV1765 | unclassified Fungi | unassigned | 2.226 | 0.323 | 2.38E-13 | |
| *Pinus* Bark | ASV0009 | unidentified Sympoventuriaceae | unassigned | 2.133 | 0.325 | 1.24E-12 | |
| *Pinus* Bark | ASV2485 | unclassified Fungi | unassigned | 2.1 | 0.318 | 0.0001 | |
| *Pinus* Bark | ASV0006 | unidentified Sympoventuriaceae | unassigned | 2.089 | 0.325 | 2.50E-12 | |
| *Pinus* Bark | ASV1089 | unclassified Fungi | unassigned | 2.058 | 0.323 | 8.43E-11 | |
| *Pinus* Bark | ASV1943 | unclassified Fungi | unassigned | 2.056 | 0.315 | 0.0012 | |
| *Pinus* Bark | ASV2586 | unidentified Fungi | unassigned | 2.015 | 0.299 | 0.0055 | |
| *Pinus* Bark | ASV2196 | unidentified Fungi | unassigned | 1.853 | 0.321 | 8.25E-09 | |
| *Pinus* Bark | ASV0754 | unidentified Capnodiales | unassigned | 1.837 | 0.306 | 0.0103 | |
| *Pinus* Bark | ASV1764 | unclassified Fungi | unassigned | 1.761 | 0.324 | 1.22E-08 | |
| *Pinus* Bark | ASV1627 | unclassified Fungi | unassigned | 1.743 | 0.321 | 4.81E-08 | |
| *Pinus* Bark | ASV2201 | unidentified Fungi | unassigned | 1.742 | 0.317 | 6.16E-08 | |
| *Pinus* Bark | ASV2440 | unclassified Eurotiomycetes | unassigned | 1.735 | 0.325 | 4.25E-08 | |
| *Pinus* Bark | ASV1091 | unclassified Fungi | unassigned | 1.716 | 0.315 | 8.94E-08 | |
| *Pinus* Bark | ASV1073 | unclassified Fungi | unassigned | 1.693 | 0.31 | 0.0065 | |
| *Pinus* Bark | ASV2198 | unidentified Fungi | unassigned | 1.687 | 0.32 | 1.69E-07 | |
| *Pinus* Bark | ASV1112 | unclassified Fungi | unassigned | 1.687 | 0.311 | 0.0009 | |
| *Pinus* Bark | ASV2301 | unidentified Chaetothyriales | unassigned | 1.686 | 0.314 | 0.0005 | |
| *Pinus* Bark | ASV1630 | unclassified Fungi | unassigned | 1.669 | 0.318 | 2.00E-07 | |
| *Pinus* Bark | ASV0339 | unclassified *Devriesia* | plant_pathogen | 1.657 | 0.303 | 0.0167 | |
| *Pinus* Bark | ASV1629 | unclassified Fungi | unassigned | 1.606 | 0.322 | 6.23E-07 | |
| *Pinus* Bark | ASV1621 | unclassified Fungi | unassigned | 1.583 | 0.322 | 9.40E-07 | |
| *Pinus* Bark | ASV2199 | unidentified Fungi | unassigned | 1.578 | 0.322 | 1.02E-06 | |
| *Pinus* Bark | ASV2230 | unclassified Fungi | unassigned | 1.569 | 0.313 | 0.0036 | |
| *Pinus* Bark | ASV1116 | unclassified Fungi | unassigned | 1.556 | 0.296 | 0.0106 | |
| *Pinus* Bark | ASV1113 | unclassified Ascomycota | unassigned | 1.547 | 0.321 | 1.68E-06 | |
| *Pinus* Bark | ASV1088 | unclassified Fungi | unassigned | 1.508 | 0.325 | 1.94E-06 | |
| *Pinus* Bark | ASV2506 | unclassified Ascomycota | animal_parasite | 1.491 | 0.315 | 0.0016 | |
| *Pinus* Bark | ASV2189 | *Exophiala bergeri* | unassigned | 1.491 | 0.303 | 0.006 | |
| *Pinus* Bark | ASV1459 | unidentified *Pseudochaetosphaeronema* | litter_saprotroph | 1.464 | 0.307 | 0.0166 | |
| *Pinus* Bark | ASV1622 | unclassified Fungi | unassigned | 1.416 | 0.32 | 9.36E-06 | |
| *Pinus* Bark | ASV1122 | unclassified Fungi | unassigned | 1.313 | 0.301 | 0.0197 | |
| *Pinus* Bark | ASV1623 | unclassified Fungi | unassigned | 1.289 | 0.318 | 6.34E-05 | |
| *Pinus* Bark | ASV2563 | unclassified Fungi | unassigned | 1.274 | 0.316 | 0.0003 | |
| *Pinus* Bark | ASV2304 | unidentified Chaetothyriales | unassigned | 1.238 | 0.318 | 0.0001 | |
| *Pinus* Bark | ASV2054 | *Helminthosporium asterinum* | plant_pathogen | 1.211 | 0.318 | 0.0002 | |
| *Pinus* Bark | ASV1376 | unclassified Fungi | unassigned | 1.162 | 0.325 | 0.0003 | |
| *Pinus* Bark | ASV1626 | unclassified Fungi | unassigned | 1.121 | 0.318 | 0.0005 | |
| *Dirinaria* | ASV1187 | *Pestalotiopsis rhododendri* | plant_pathogen | -1.142 | 0.315 | 0.0003 | |
| *Dirinaria* | ASV2123 | unidentified *Phylliscum* | litter_saprotroph | -1.16 | 0.322 | 0.0097 | |
| *Dirinaria* | ASV0809 | unclassified Capnodiales | unassigned | -1.231 | 0.267 | 0.0203 | |
| *Dirinaria* | ASV0121 | unclassified Dothideomycetes | unassigned | -1.24 | 0.282 | 0.0137 | |
| *Dirinaria* | ASV2227 | *Veronaea compacta* | plant_pathogen | -1.296 | 0.304 | 0.0173 | |
| *Dirinaria* | ASV0545 | unclassified Fungi | unassigned | -1.323 | 0.308 | 0.01 | |
| *Dirinaria* | ASV0612 | unclassified Dothideomycetes | unassigned | -1.327 | 0.318 | 3.94E-05 | |
| *Dirinaria* | ASV2176 | unclassified Fungi | unassigned | -1.384 | 0.317 | 1.77E-05 | |
| *Dirinaria* | ASV0105 | unclassified Capnodiales | unassigned | -1.439 | 0.311 | 0.0004 | |
| *Dirinaria* | ASV2379 | unclassified Fungi | unassigned | -1.576 | 0.304 | 0.0124 | |
| *Dirinaria* | ASV0305 | unidentified *Devriesia* | plant_pathogen | -1.619 | 0.308 | 0.0066 | |
| *Dirinaria* | ASV2339 | unclassified Fungi | unassigned | -1.63 | 0.307 | 0.0053 | |
| *Dirinaria* | ASV1158 | *Tolypocladium pustulatum* | plant_pathogen | -1.64 | 0.312 | 0.0037 | |
| *Dirinaria* | ASV0611 | unclassified Dothideomycetes | unassigned | -1.687 | 0.315 | 0.0001 | |
| *Dirinaria* | ASV0763 | unclassified Capnodiales | unassigned | -1.822 | 0.306 | 0.0081 | |
| *Dirinaria* | ASV0087 | unclassified Capnodiales | unassigned | -1.929 | 0.318 | 0.0001 | |
| *Dirinaria* | ASV0103 | unclassified Capnodiales | unassigned | -1.932 | 0.318 | 7.76E-05 | |
| *Dirinaria* | ASV1156 | *Tolypocladium pustulatum* | plant_pathogen | -1.957 | 0.315 | 0.0009 | |
| *Dirinaria* | ASV0114 | unclassified Dothideomycetes | unassigned | -2.463 | 0.322 | 1.92E-05 | |
| *Dirinaria* | ASV0028 | unclassified Capnodiales | unassigned | -2.753 | 0.303 | 0.0054 | |

Log_2_ FC^*^ Log_2_ fold change, LSE^*^ Log_2_ fold change standard error
