# Supplementary material for: Fungal community inside lichen: a curious case of sparse diversity and high modularity
Source: Environ Microbiome. 2023 Oct 3;18:73. doi: 10.1186/s40793-023-00531-8 (PMC10548754; doi:10.1186/s40793-023-00531-8)
Supplement: Supplementary file 1 — Additional file 1. Fig. S1 Community similarity ordination based on geographical distance. The ordinations are visualized in two approaches: a non-metric multidimensional scaling and b principal co-ordinates analysis based on Brat-Curtis distance. Ellipses indicate the 95% confidence interval of group variance. c PCoA plots showing ELF community variability between the two host lichens from same site. Fig. S2 Centrality indices of the fungal communities. a Betweenness and Closeness indicate vectors containing the betweenness and closeness of each node. b InDegree and OutDegree indicate vectors containing the inward and outward degree of each node. c Expected influences indicates the sums of incoming or outgoing edge weights connected to a node. Mean ± SEM, ****p < 0.001, **p < 0.01, ns not significant. [file 40793_2023_531_MOESM1_ESM.zip › Table S3.docx]

**Table S3** Result of a chi-squared test of the pairwise comparison between the endolichenic fungal communities of *Parmotrema* and epiphytic fungal communities

| **Abundant in** | **ASV no.** | **Taxa** | **Trophic Mode** | **Log_2_ FC^*^** | **LSE^*^** | ***p* value** |
| --- | --- | --- | --- | --- | --- | --- |
| *Pinus* Bark | ASV0011 | unidentified Sympoventuriaceae | unassigned | 2.803 | 0.373 | 2.47E-17 |
| *Pinus* Bark | ASV0009 | unidentified Sympoventuriaceae | unassigned | 2.444 | 0.377 | 6.71E-14 |
| *Pinus* Bark | ASV0006 | unidentified Sympoventuriaceae | unassigned | 2.405 | 0.379 | 2.23E-14 |
| *Pinus* Bark | ASV0008 | unidentified Sympoventuriaceae | unassigned | 2.319 | 0.379 | 9.40E-14 |
| *Pinus* Bark | ASV0005 | unidentified Sympoventuriaceae | unassigned | 2.307 | 0.379 | 7.43E-13 |
| *Pinus* Bark | ASV1841 | unclassified Basidiomycota | unassigned | 2.181 | 0.38 | 7.06E-13 |
| *Pinus* Bark | ASV1839 | unclassified Basidiomycota | unassigned | 2.17 | 0.38 | 4.80E-12 |
| *Pinus* Bark | ASV0548 | unclassified Fungi | unassigned | 2.134 | 0.372 | 0.0053 |
| *Pinus* Bark | ASV1838 | unclassified Basidiomycota | unassigned | 2.104 | 0.38 | 1.48E-12 |
| *Pinus* Bark | ASV2435 | unclassified Fungi | unassigned | 2.098 | 0.373 | 0.0034 |
| *Pinus* Bark | ASV1840 | unclassified Basidiomycota | unassigned | 2.044 | 0.38 | 3.35E-11 |
| *Pinus* Bark | ASV0796 | unclassified Capnodiales | unassigned | 2.018 | 0.37 | 0.0069 |
| *Pinus* Bark | ASV0542 | unclassified Capnodiales | unassigned | 1.98 | 0.372 | 0.004 |
| *Pinus* Bark | ASV2485 | unclassified Fungi | unassigned | 1.971 | 0.373 | 0.0016 |
| *Pinus* Bark | ASV1766 | unclassified Basidiomycota | unassigned | 1.97 | 0.376 | 7.66E-09 |
| *Pinus* Bark | ASV0543 | unclassified Capnodiales | unassigned | 1.946 | 0.371 | 0.0056 |
| *Pinus* Bark | ASV1943 | unclassified Fungi | unassigned | 1.944 | 0.37 | 0.0069 |
| *Pinus* Bark | ASV1089 | unclassified Fungi | unassigned | 1.905 | 0.378 | 6.99E-08 |
| *Pinus* Bark | ASV2563 | unclassified Fungi | unassigned | 1.903 | 0.372 | 0.0024 |
| *Pinus* Bark | ASV0802 | unclassified Capnodiales | unassigned | 1.901 | 0.371 | 0.0062 |
| *Pinus* Bark | ASV2482 | unclassified Fungi | unassigned | 1.86 | 0.374 | 0.0012 |
| *Pinus* Bark | ASV1765 | unclassified Fungi | unassigned | 1.83 | 0.378 | 6.96E-08 |
| *Pinus* Bark | ASV1763 | unclassified Basidiomycota | unassigned | 1.805 | 0.377 | 1.46E-07 |
| *Pinus* Bark | ASV1627 | unclassified Fungi | unassigned | 1.802 | 0.375 | 7.74E-07 |
| *Pinus* Bark | ASV1088 | unclassified Fungi | unassigned | 1.792 | 0.378 | 5.15E-07 |
| *Pinus* Bark | ASV2196 | unidentified Fungi | unassigned | 1.743 | 0.376 | 1.83E-06 |
| *Pinus* Bark | ASV1376 | unclassified Fungi | unassigned | 1.726 | 0.377 | 1.85E-06 |
| *Pinus* Bark | ASV0539 | unclassified Capnodiales | unassigned | 1.708 | 0.377 | 0.0005 |
| *Pinus* Bark | ASV1764 | unclassified Fungi | unassigned | 1.662 | 0.378 | 1.47E-06 |
| *Pinus* Bark | ASV1622 | unclassified Fungi | unassigned | 1.65 | 0.374 | 8.02E-06 |
| *Pinus* Bark | ASV1630 | unclassified Fungi | unassigned | 1.645 | 0.372 | 8.96E-06 |
| *Pinus* Bark | ASV1091 | unclassified Fungi | unassigned | 1.642 | 0.369 | 9.80E-06 |
| *Pinus* Bark | ASV1112 | unclassified Fungi | unassigned | 1.62 | 0.365 | 0.0058 |
| *Pinus* Bark | ASV2301 | unidentified Chaetothyriales | unassigned | 1.614 | 0.368 | 0.0037 |
| *Pinus* Bark | ASV2236 | unclassified Fungi | unassigned | 1.606 | 0.376 | 1.27E-05 |
| *Pinus* Bark | ASV0533 | unclassified Dothideomycetes | unassigned | 1.6 | 0.38 | 5.79E-06 |
| *Pinus* Bark | ASV2440 | unclassified Eurotiomycetes | unassigned | 1.598 | 0.38 | 5.49E-06 |
| *Pinus* Bark | ASV2198 | unidentified Fungi | unassigned | 1.598 | 0.375 | 1.51E-05 |
| *Pinus* Bark | ASV2230 | unclassified Fungi | unassigned | 1.597 | 0.368 | 0.0077 |
| *Pinus* Bark | ASV1382 | unclassified Fungi | unassigned | 1.591 | 0.377 | 1.45E-05 |
| *Pinus* Bark | ASV0986 | unclassified Dothideomycetes | unassigned | 1.582 | 0.371 | 0.0022 |
| *Pinus* Bark | ASV0983 | unclassified Capnodiales | unassigned | 1.581 | 0.377 | 1.65E-05 |
| *Pinus* Bark | ASV0531 | unclassified Ascomycota | unassigned | 1.579 | 0.376 | 0.0009 |
| *Pinus* Bark | ASV2201 | unidentified Fungi | unassigned | 1.535 | 0.371 | 3.08E-05 |
| *Pinus* Bark | ASV1000 | unclassified Dothideomycetes | unassigned | 1.527 | 0.378 | 2.96E-05 |
| *Pinus* Bark | ASV2232 | unclassified Fungi | unassigned | 1.524 | 0.377 | 3.42E-05 |
| *Pinus* Bark | ASV2234 | unclassified Ascomycota | unassigned | 1.51 | 0.376 | 4.21E-05 |
| *Pinus* Bark | ASV2231 | unclassified Ascomycota | unassigned | 1.498 | 0.377 | 4.76E-05 |
| *Pinus* Bark | ASV1381 | unclassified Fungi | unassigned | 1.477 | 0.377 | 5.91E-05 |
| *Pinus* Bark | ASV1113 | unclassified Ascomycota | unassigned | 1.465 | 0.376 | 7.40E-05 |
| *Pinus* Bark | ASV2510 | unclassified Ascomycota | unassigned | 1.458 | 0.37 | 0.0061 |
| *Pinus* Bark | ASV2506 | unclassified Ascomycota | unassigned | 1.445 | 0.368 | 0.0075 |
| *Pinus* Bark | ASV1767 | unclassified Fungi | unassigned | 1.443 | 0.379 | 3.31E-05 |
| *Pinus* Bark | ASV0997 | unclassified Dothideomycetes | unassigned | 1.38 | 0.369 | 0.0075 |
| *Pinus* Bark | ASV1626 | unclassified Fungi | unassigned | 1.317 | 0.372 | 0.0004 |
| *Pinus* Bark | ASV2199 | unidentified Fungi | unassigned | 1.3 | 0.377 | 0.0004 |
| *Pinus* Bark | ASV0632 | unclassified Dothideomycetes | unassigned | 1.22 | 0.372 | 0.0011 |
| *Pinus* Bark | ASV1842 | unclassified Basidiomycota | unassigned | 1.218 | 0.377 | 0.0001 |
| *Pinus* Bark | ASV1762 | unclassified Basidiomycota | unassigned | 1.198 | 0.379 | 0.0007 |
| *Pinus* Bark | ASV2054 | *Helminthosporium asterinum* | plant_pathogen | 1.166 | 0.373 | 0.0018 |
| *Pinus* Bark | ASV0992 | unclassified Fungi | unassigned | 1.108 | 0.371 | 0.0031 |
| *Pinus* Bark | ASV2200 | unidentified Fungi | unassigned | 1.094 | 0.369 | 0.0034 |
| *Pinus* Bark | ASV2188 | *Exophiala bergeri* | animal_parasite | 1.071 | 0.369 | 0.0041 |
| *Pinus* Bark | ASV0426 | *Devriesia strelitziicola* | plant_pathogen | 1.016 | 0.372 | 0.0067 |
| *Parmotrema* | ASV2178 | unclassified Fungi | unassigned | -1 | 0.347 | 0.0042 |
| *Parmotrema* | ASV1344 | unclassified Fungi | unassigned | -1.017 | 0.358 | 0.0048 |
| *Parmotrema* | ASV1185 | *Pestalotiopsis rhododendri* | plant_pathogen | -1.064 | 0.377 | 0.0056 |
| *Parmotrema* | ASV0826 | unclassified Capnodiales | unassigned | -1.116 | 0.352 | 0.0017 |
| *Parmotrema* | ASV2547 | unclassified Fungi | unassigned | -1.186 | 0.354 | 0.0009 |
| *Parmotrema* | ASV1225 | unclassified Fungi | unassigned | -1.247 | 0.357 | 0.0006 |
| *Parmotrema* | ASV1563 | unidentified Fungi | unassigned | -1.263 | 0.357 | 0.0005 |
| *Parmotrema* | ASV1196 | *Pestalotiopsis rhododendri* | plant_pathogen | -1.368 | 0.348 | 0.0003 |
| *Parmotrema* | ASV2123 | unidentified *Phylliscum* | litter_saprotroph | -1.384 | 0.372 | 0.0003 |
| *Parmotrema* | ASV2620 | unclassified Fungi | unassigned | -1.397 | 0.36 | 0.0001 |
| *Parmotrema* | ASV1192 | *Pestalotiopsis rhododendri* | plant_pathogen | -1.417 | 0.373 | 0.0002 |
| *Parmotrema* | ASV0974 | unidentified Capnodiales | unassigned | -1.519 | 0.38 | 9.27E-05 |
| *Parmotrema* | ASV0087 | unclassified Capnodiales | unassigned | -1.64 | 0.357 | 0.0001 |
| *Parmotrema* | ASV1580 | unclassified Fungi | unassigned | -1.659 | 0.377 | 1.64E-05 |
| *Parmotrema* | ASV2344 | unclassified Fungi | unassigned | -1.659 | 0.372 | 0.0009 |
| *Parmotrema* | ASV1967 | unclassified Basidiomycota | unassigned | -1.767 | 0.356 | 0.0034 |
| *Parmotrema* | ASV1186 | *Pestalotiopsis rhododendri* | plant_pathogen | -1.772 | 0.377 | 4.34E-06 |
| *Parmotrema* | ASV0970 | unidentified Capnodiales | unassigned | -1.821 | 0.379 | 2.52E-06 |
| *Parmotrema* | ASV1195 | *Pestalotiopsis rhododendri* | plant_pathogen | -1.822 | 0.349 | 0.0007 |
| *Parmotrema* | ASV1187 | *Pestalotiopsis rhododendri* | plant_pathogen | -1.824 | 0.367 | 1.16E-06 |
| *Parmotrema* | ASV1341 | unclassified Fungi | unassigned | -1.851 | 0.336 | 0.0079 |
| *Parmotrema* | ASV1584 | unclassified Fungi | unassigned | -1.902 | 0.352 | 0.0017 |
| *Parmotrema* | ASV1577 | unclassified Fungi | unassigned | -1.967 | 0.378 | 3.51E-07 |
| *Parmotrema* | ASV2125 | unidentified *Phylliscum* | litter_saprotroph | -1.975 | 0.366 | 0.0001 |
| *Parmotrema* | ASV2343 | unclassified Fungi | unassigned | -1.998 | 0.372 | 1.63E-07 |
| *Parmotrema* | ASV1540 | unidentified Fungi | unassigned | -2.014 | 0.371 | 2.86E-05 |
| *Parmotrema* | ASV0084 | unclassified Capnodiales | unassigned | -2.04 | 0.335 | 0.0021 |
| *Parmotrema* | ASV1368 | unclassified Fungi | unassigned | -2.062 | 0.357 | 0.0006 |
| *Parmotrema* | ASV1576 | unclassified Fungi | unassigned | -2.098 | 0.355 | 0.0008 |
| *Parmotrema* | ASV1194 | *Pestalotiopsis rhododendri* | plant_pathogen | -2.128 | 0.371 | 2.09E-08 |
| *Parmotrema* | ASV1544 | unidentified Fungi | unassigned | -2.18 | 0.374 | 1.39E-08 |
| *Parmotrema* | ASV1183 | *Pestalotiopsis rhododendri* | plant_pathogen | -2.243 | 0.372 | 3.55E-09 |
| *Parmotrema* | ASV0729 | *Neodevriesia lagerstroemiae* | plant_pathogen | -2.244 | 0.364 | 8.11E-06 |
| *Parmotrema* | ASV0117 | unclassified Capnodiales | unassigned | -2.288 | 0.371 | 9.31E-07 |
| *Parmotrema* | ASV1799 | *Clitopilus hobsonii* | litter_saprotroph | -2.46 | 0.369 | 4.41E-05 |
| *Parmotrema* | ASV1583 | unclassified Fungi | unassigned | -2.47 | 0.371 | 7.98E-11 |
| *Parmotrema* | ASV1582 | unclassified Fungi | unassigned | -2.471 | 0.376 | 1.49E-10 |
| *Parmotrema* | ASV1801 | *Clitopilus hobsonii* | litter_saprotroph | -2.52 | 0.362 | 0.0002 |
| *Parmotrema* | ASV1578 | unclassified Fungi | unassigned | -2.584 | 0.368 | 1.29E-06 |
| *Parmotrema* | ASV1581 | unclassified Fungi | unassigned | -2.631 | 0.376 | 1.02E-11 |
| *Parmotrema* | ASV1567 | unidentified Fungi | unassigned | -2.743 | 0.359 | 0.0002 |
| *Parmotrema* | ASV1574 | unclassified Fungi | unassigned | -2.751 | 0.37 | 6.90E-13 |

Log_2_ FC^*^ Log_2_ fold change, LSE^*^ Log_2_ fold change standard error
