# Supplementary figures and images for: Fungal community inside lichen: a curious case of sparse diversity and high modularity
Source: Environ Microbiome. 2023 Oct 3;18:73. doi: 10.1186/s40793-023-00531-8 (PMC10548754; doi:10.1186/s40793-023-00531-8)

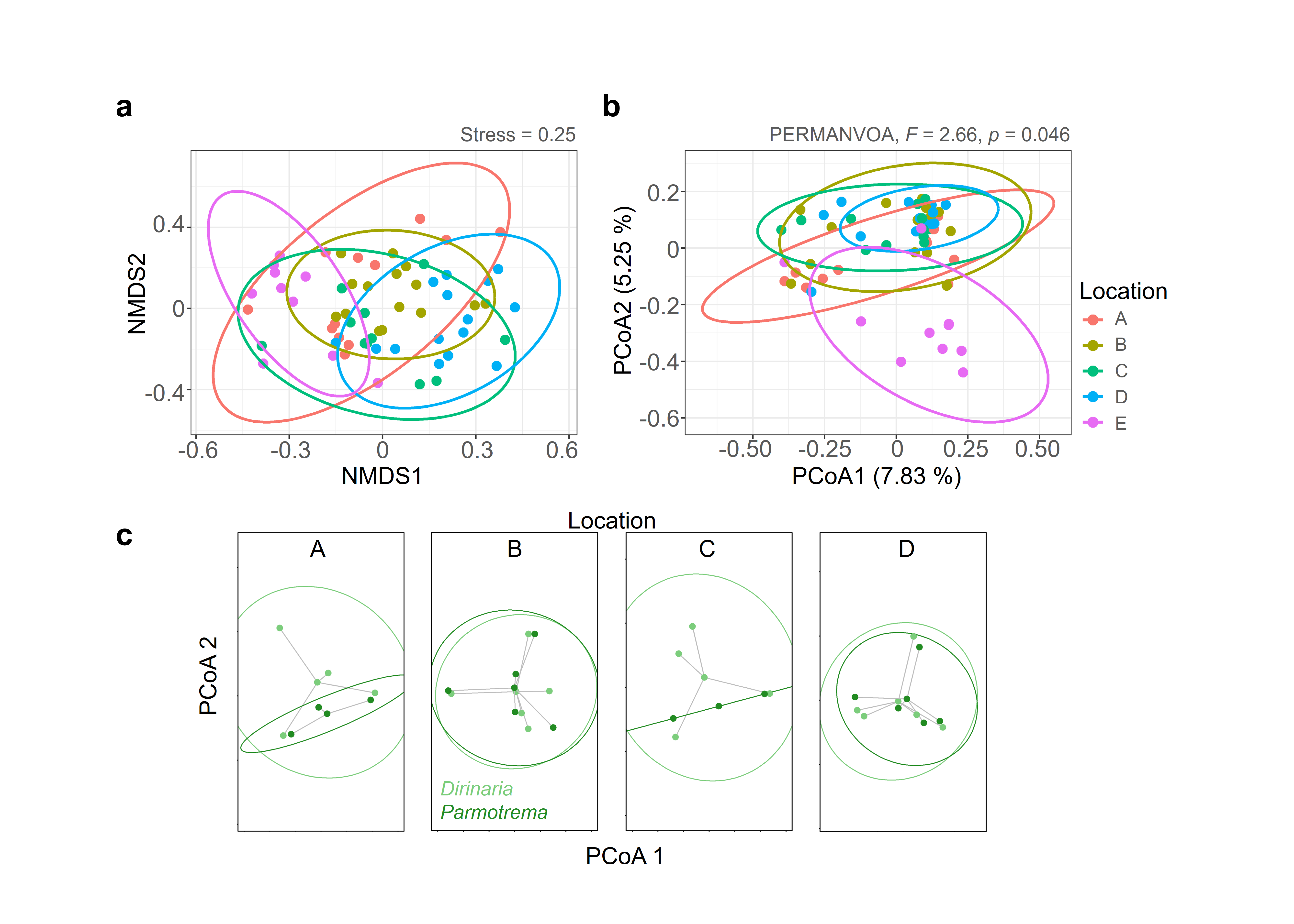

Supplement: Supplementary file 1 — Additional file 1. Fig. S1 Community similarity ordination based on geographical distance. The ordinations are visualized in two approaches: a non-metric multidimensional scaling and b principal co-ordinates analysis based on Brat-Curtis distance. Ellipses indicate the 95% confidence interval of group variance. c PCoA plots showing ELF community variability between the two host lichens from same site. Fig. S2 Centrality indices of the fungal communities. a Betweenness and Closeness indicate vectors containing the betweenness and closeness of each node. b InDegree and OutDegree indicate vectors containing the inward and outward degree of each node. c Expected influences indicates the sums of incoming or outgoing edge weights connected to a node. Mean ± SEM, ****p < 0.001, **p < 0.01, ns not significant. [file 40793_2023_531_MOESM1_ESM.zip › Figure S1.TIF]

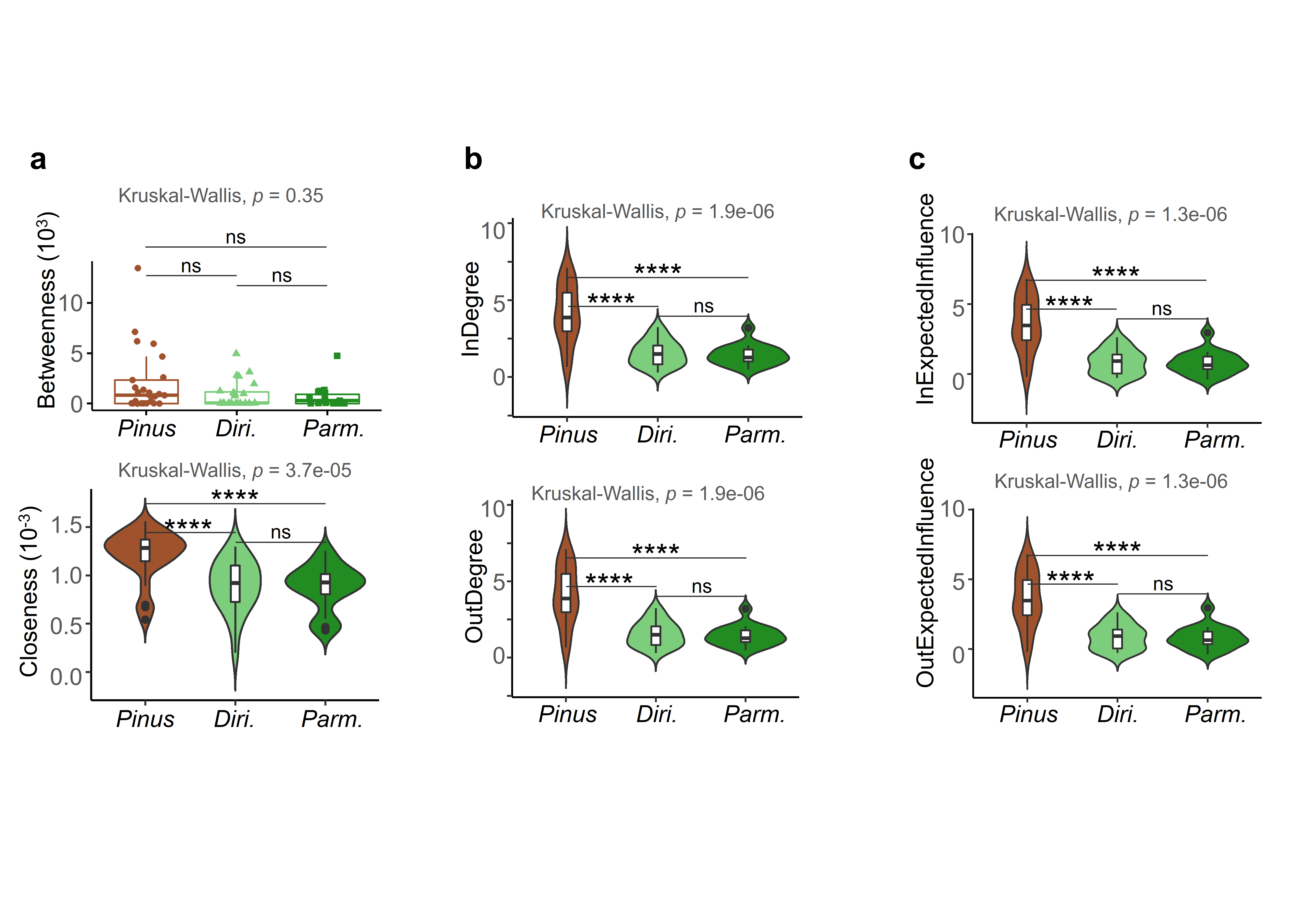

Supplement: Supplementary file 1 — Additional file 1. Fig. S1 Community similarity ordination based on geographical distance. The ordinations are visualized in two approaches: a non-metric multidimensional scaling and b principal co-ordinates analysis based on Brat-Curtis distance. Ellipses indicate the 95% confidence interval of group variance. c PCoA plots showing ELF community variability between the two host lichens from same site. Fig. S2 Centrality indices of the fungal communities. a Betweenness and Closeness indicate vectors containing the betweenness and closeness of each node. b InDegree and OutDegree indicate vectors containing the inward and outward degree of each node. c Expected influences indicates the sums of incoming or outgoing edge weights connected to a node. Mean ± SEM, ****p < 0.001, **p < 0.01, ns not significant. [file 40793_2023_531_MOESM1_ESM.zip › Figure S2.TIF]
